# Clinical Results of a Brindley Procedure: Sacral Anterior Root Stimulation in Combination with a Rhizotomy of the Dorsal Roots

**DOI:** 10.1155/2011/709708

**Published:** 2011-06-22

**Authors:** F. M. J. Martens, J. P. F. A. Heesakkers

**Affiliations:** Department of Urology (659), Radboud University Nijmegen Medical Centre, P. O. Box 9101, 6500 HB Nijmegen, The Netherlands

## Abstract

The Brindley procedure consists of a stimulator for sacral anterior-root stimulation and a rhizotomy of the dorsal sacral roots to abolish neurogenic detrusor overactivity. Stimulation of the sacral anterior roots enables micturition, defecation, and erections. This overview discusses the technique, selection of patients and clinical results of the Brindley procedure. The Brindley procedure is suitable for a selected group of patients with complete spinal cord injury and detrusor overactivity. Overall, the Brindley procedure shows good clinical results and improves quality of life. However, to remain a valuable treatment option for the future, the technique needs some adequate changes to enable analysis of the implanted parts, to improve revision techniques of the implanted parts, and to abolish the sacral dorsal rhizotomy.

## 1. Introduction

The Brindley procedure consists of a stimulator for sacral anterior-root stimulation (SARS) and a rhizotomy of the dorsal sacral roots to abolish neurogenic detrusor overactivity. Stimulation of the sacral anterior roots enables micturition, defecation, and erections. The Brindley procedure is suitable for a selected group of skeletally mature patients with complete spinal cord injury and detrusor overactivity, who do not respond adequately to conservative treatments of the detrusor overactivity. Patients with severe autonomic dysreflexia or detrusor-external sphincter dyssynergia will benefit especially from the dorsal rhizotomy. Patients with incomplete injury will lose their sensory function due to the dorsal rhizotomy and have the risk to experience pain sensation during stimulation due to an incomplete rhizotomy. The technique, selection of patients, and clinical results are discussed in this overview.

## 2. Brindley Stimulator

The Brindley system is composed of an external and implanted part. The implanted part consists of electrodes, connecting cables, and a receiver block. Patients have to position an external stimulating device on the skin over the implanted receiver to evoke stimuli. The receiver does not have a battery. Electrical stimuli are evoked by radiofrequency waves. With the availability of separate stimulation of the sacral levels and various stimulation settings, it is possible to set various stimulation programs to optimize micturition, defecation, and penile erections.

A tripolar electrode cuff is used for intradural stimulation of the sacral anterior roots. A three-channel implant is composed of two books. The upper book contains three parallel slots for S3 (one slot) and S2 roots (two slots at one channel), and the lower contains one slot for S4 roots. Each slot contains one cathode in the centre and an anode at each of the two ends to avoid stimulation of tissue structures outside the slot. The two-channel implant allows stimulation of two root levels or sets of root levels. The four-channel implant has the same configuration as the three-channel implant but allows independent stimulation of four sets of roots. The choice for the number of channels depends on the number of different rootlet combinations that have to be stimulated. Each channel is connected to the subcutaneous receiver block by a silicone-coated cable.

Extradural electrodes are used in patients in whom intradural electrodes could not be placed due to, for example, arachnoiditis or a previous intradural electrode implantation that failed. Some centres prefer to use extradural electrodes primarily for nearly all patients. The extradural implant has three helical electrodes at its end, which are also configured with a cathode between two anodes.

## 3. Poststimulus Voiding

**Figure 1:**
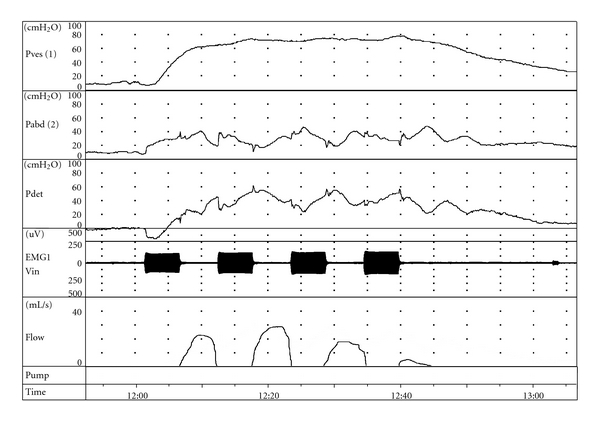
Example of poststimulus voiding using a Brindley stimulator. The three upper traces show the intravesical (Pves), intra-abdominal (Pabd), and detrusor (Pdet) pressures during stimulation with a Brindley stimulator. The increase in EMG signal reflects the activation of the stimulus during 5 seconds. Stimulation is activated every 12 seconds. The intermittent stimulation pattern allows the urethral sphincter to relax while the detrusor pressure remains elevated. This results in an intermittent flow pattern.

Most of the small diameter parasympathetic efferent nerve fibres for innervation of the bladder are located in the sacral anterior roots (S2–S4/5). Small-diameter nerve fibres need a higher stimulus for their excitation than large-diameter fibres. Consequently, electrical stimulation of the anterior roots for detrusor contractions also causes contraction of the urethral sphincter due to stimulation of somatic large-diameter nerve fibres. This prevents emptying of the bladder. To overcome this problem, poststimulus voiding is used. The time to relax of striated muscles of the urethral sphincter is shorter than the relaxation time of smooth muscles of the detrusor. When intermittent stimulation pulse trains are applied, the difference in muscle relaxation time can be used to achieve a sustained detrusor muscle contraction with intervals of urethral sphincter relaxation (Figure 1). These intervals in between stimulations allow a decrease of the urethral sphincter pressure while a high intravesical pressure remains. This results in poststimulus voiding with an intermittent pattern of the micturition flow. A comparable mechanism has been used for defecation.

## 4. Dorsal Rhizotomy of the Sacral Nerves

Sauerwein structurally expanded SARS with a dorsal rhizotomy (deafferentation) of sacral roots S2 till S5 [1]. A dorsal rhizotomy is important because it suppresses neurogenic detrusor overactivity and detrusor-external sphincter dyssynergia [1, 2]. This results in a low-pressure bladder without reflex contractions of the detrusor and subsequently continence. Moreover, it reduces autonomic dysreflexia [2, 3]. Therefore, a dorsal rhizotomy can also be applied in combination with intermittent catheterization to empty the bladder without implantation of a Brindley stimulator [3].

## 5. Patient Selection

Patients need to have intact efferent nerve pathways to the bladder and a bladder that is able to contract. Contractions of at least 50 cm H_2_O in males or 30 cm H_2_O in females need to be present during filling cystometry [4]. If no sufficient spontaneous contraction occurs, suitable patients can be selected by rectal stimulation according to electroejaculation procedures or direct needle stimulation of the sacral roots to provoke bladder contractions.

Preoperative magnetic resonance imaging is used to exclude arachnoiditis at the level of the conus and cauda equine or other neurological disorder of the spinal cord. Patients with active or previous arachnoiditis are not suitable for intradural electrode implantation.

## 6. Implantation

A laminectomy from L3-L4 to S1-S2 is done for an intradural rhizotomy and intradural implantation of the electrode cuff. The dura and arachnoid are opened at midline to expose the sacral nerve roots. The anterior and dorsal components of the roots, especially relevant anterior roots for micturition, can be identified intradurally by electrical stimulation of these components while monitoring the effects on detrusor activity, blood pressure, and somatomotor responses. A rhizotomy of the identified dorsal sacral roots is done. The anterior sacral roots are positioned into the electrode cuff. The connecting cables are subcutaneously tunnelled to a subcutaneous pocket for the receiver.

Implantation of extradural electrodes requires a laminectomy from L5-S1 to S3-S4. The dorsal rhizotomy is done at the level of the ganglia of S2-S5. Electrical stimulation tests are used to identify the anterior and dorsal components of the sacral roots. The extradural electrode is implanted and fixated to the nerve using a strip of silicone rubber sheet which is sewn to itself and surrounds the nerve. The connecting cables and the receiver are implanted the same way as the intradural procedure.

## 7. Clinical Results

**Figure 2:**
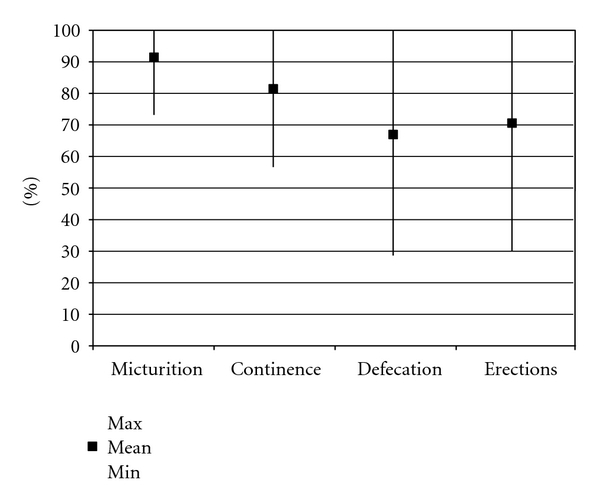
Results of the Brindley procedure on micturition, continence, defecation, and erections are summarised.

Table 1 shows an overview of publications on the clinical results of the Brindley procedure [3–20]. These results comprise both the Brindley stimulator, which enables stimulation for micturition, defecation, and erections, and the dorsal rhizotomy to achieve continence. The use of the Brindley procedure for micturition and defecation, and the ability to evoke erections are summarised in Figure 2, including urinary continence rates. No accumulation of results is possible due to the overlap of results of several reports, especially the multicentre reports. 

The Brindley stimulator is used for micturition in 73% to 100% of patients during followup. These are considerable percentages, but it should be noted that this includes patients who use additional methods to empty their bladder. Additional methods comprise intermittent catheterization, abdominal straining (Valsalva manoeuvre), abdominal compression (Credé manoeuvre), or suprapubic tapping for reflex contractions. Stimulation that is not always completely successful can be found back in the percentages of patients that have less than 50 mL residual urine after stimulation for micturition. These percentages are lower than the percentages of patients that use the stimulator for micturition. Overall, the percentages of patients having urinary tract infections and the frequency of urinary tract infections decrease after the Brindley procedure compared to the preoperative treatment.

The Brindley stimulator is used for defecation in 29% to 100% of patients in different degrees. Not all patients achieve complete evacuation of defecation using only stimulation. Some patients need laxatives in addition to prevent constipation or enable defecation. Many patients only use the stimulator to get the defecation into the rectum, to enable digital evacuation.

Erections can be evoked in a substantial number of patients, but results vary considerably. This can be explained by the relatively low number of patients that actually use the stimulator to evoke erections for sexual intercourse (0–32%), due to qualitatively inadequate erections for sexual intercourse or deterioration of the stimulation effect over time.

Autonomic dysreflexia mostly decreased after the Brindley procedure as a result of the dorsal rhizotomy. Only a few studies reported stimulation-induced autonomic dysreflexia. 

Continence is achieved in 57% to 100% of patients, and bladder capacity increased. However, continence is not only achieved by a dorsal rhizotomy. Results on continence also included additional treatments, like anticholinergics and stress incontinence surgery.

## 8. Discussion

The ultimate treatment of neurogenic disorders of the lower urinary tract would be resolvement of the neurogenic disorder that causes the bladder problems to restore the innervation of the bladder. As long as this causal treatment is not available, symptomatic treatment options are required.

Intravesical Botulinum toxin A injections are an evolving option in the current treatment arsenal. At the time when this paper was written, approval for urological application was expected within short time. However, the Brindley procedure has several advantages for suitable patients compared to Botulinum toxin A in combination with intermittent catheterization, especially if not only the urological properties of the treatments are taken into account. Spinal cord injury comprises a variety of coherent, physical problems. Therefore, management of multiple organ dysfunctions should be advocated. The Brindley procedure does not only enable continence and micturition, but also complete defecation or improvement of defecation pattern, penile erections, and reduction of autonomic dysreflexia and spasms. Patients become less dependent because they do not need assistance for intermittent catheterization anymore and can empty their bladder whereever and whenever. When the treatment options are discussed with a patient, this more extensive application of the Brindley procedure should be mentioned.

The Brindley procedure generally shows good clinical results for restoration of function in spinal cord injury patients with multiple organ dysfunction, including bladder, bowel, and erectile dysfunction. Moreover, the Brindley procedure improves quality of life [20, 21]. However, it is not a procedure that is easy to apply in clinical practice. Firstly, not every patient is suited for the procedure and the success depends on selection of appropriate patients. Prerequisites are a complete spinal cord lesion since neurostimulation can cause pain in incomplete spinal cord lesions, an intact sacral motor neuron pathway enabling stimulation of the bladder, and a detrusor muscle that is capable to contract on stimulation. Secondly, a dorsal rhizotomy and implantation of a Brindley stimulator is complex and not a routine procedure for urologists and should be reserved for specialized centres. Thirdly, the technique is also prone to failures, including the external and implanted components. Analysis of the external components is easy to apply. Currently, a straightforward solution for analysis and revision of the implanted system without major surgery is not available in every country. This can be explained by national legislation with respect to certain aspects of the surgical procedure for revision of the implant, like burning the insulation of the implanted electrode cables. This excludes these patients from the thorough analysis of the implanted components and revision surgery to restore function of their stimulator. Nowadays, most patients have become increasingly familiar with intermittent catheterization and bowel rinsing. They accept the dysfunction of the stimulator more frequently because they remain continent due to their dorsal rhizotomy in combination with controlled emptying of their bladder and bowels.

A main issue for patients who consider a Brindley procedure is the irreversibility of the rhizotomy, and the possibility that future treatment options are not within reach anymore. Although SARS can restore penile erections after a rhizotomy, qualitative useful stimulation of erections is not possible in a substantial number of patients. Therefore, the dorsal rhizotomy should be replaced by a less invasive procedure to abolish detrusor overactivity. Continuous or conditional neuromodulation could be one of the solutions [22, 23]. Sacral posterior- and anterior- root stimulation combines neuromodulation and SARS without a rhizotomy of the dorsal roots for micturition. These new developments are, however, not generally introduced as a standard treatment. Sacral posterior and anterior root stimulation effectively suppress DO but do not result in complete emptying in all patients due to persisting detrusor-external sphincter dyssynergia [24]. This requires development of techniques that prevent backward stimulation when the anterior roots are stimulated to enable selective detrusor stimulation, like selective anodal block and high-frequency block [25–28].

## 9. Conclusion

The Brindley procedure shows good clinical results and improves quality of life. However, to remain a valuable treatment option for the future, the technique needs some adequate changes to enable analysis of the implanted parts, to improve revision techniques of the implanted parts, and to abolish the sacral dorsal rhizotomy.

## Figures and Tables

**Table 1:** Publications on clinical results of the Brindley procedure.

Author	Patients in followup	Rhizotomy (intradural/ extradural)	Implantation (intradural/ extradural)	Followup (years)	Autonomic dysreflexia (before/after)	Use for voiding (% of patients)	Continence (% of patients)	Bladder capacity (% of increase)	Residual urine (% of patients)	UTI incidence (% of patients or incidence/year)	Use for defecation (% of patients)	Use for erections (% of males)
Brindley et al. [4]	38 ♂ 12 ♀	17/0 (S2 and/or S3)	50/0	1–9	0/1 (during stimulation)	86%	62%	—	80% <60 mL	—	—	68%
Madersbacher et al. [12]	1 ♂ 6 ♀	7/0	7/0	—	—(1 during stimulation)	100%	100%	122%	100% <40 mL	After 0%	29%	100%
Robinson et al. [14]	20 ♂ 2 ♀	—	—	—	—	73%	68%	—	—	—	—	30% (0% of ♂ used stimulation for sexual intercourse)
MacDonagh et al. [11]	9 ♂ 3 ♀	9/0	12/0	2.2	—	100%	—	—	—	—	50% complete emptying with stimulation	—
Sauerwein et al. [16]	5 ♂ 6 ♀	0/12	0/12	—	—	82%	64%	—	100% <50 mL	—	—	—
Van Kerrebroeck et al. [19]	90 ♂ 94 ♀ ∗∗	—	166/18	—	26/10	92%	86%	—	82%<30 mL	Before 88% After 17%	70%	74% (32% of ♂ used stimulation for sexual intercourse)
Madersbacher et al. [13]	8 ♂ 22 ♀	—	27/4	—	—	97%	93%	—	90% <50 mL	—	—	—
Sarrias et al. [15]	1 ♂ 6 ♀	7/0	0/7	—	—	100%	100%	—	100% <50 mL	—	100%	—
Brindley [6]	271 ♂ 229 ♀ ∗∗	—	≤477/≥23	4	—(3 during stimulation)	86%	—	—	—	—	—	—
Van Kerrebroeck et al. [3]	29 ♂ 18 ♀	47/0	47/0	3.5	7/5 (2 during stimulation)	96%	91%	—	87%<50 mL	Before 4.2/year After 1.4/year	87%	62% (21% of ♂ used stimulation for sexual intercourse)
Schurch et al. [17]	3 ♂ 7 ♀	10/0	10/0	3.4	6/6 (during stimulation)	100%	80%	213%	100% <50 mL	Before 80% After 60%	—	—
Egon et al. [8]	68 ♂ 28 ♀	−/−	90/9	5.4 ♂ 5.8 ♀	22/0	90%	89%	134% ♂ 375% ♀ (range 300–600)	86% <50 mL	Before 100% After 31%	55% (41% of these complete evacuation with stimulation alone)	75% (26% of ♂ used stimulation for sexual intercourse)
V/d Aa et al. [18]	33 ♂ 4 ♀	37/0	37/0	0.3–12	—	100%	84%	—	73% <30 mL	—	73%	88%
Bauchet et al. [5]	6 ♂ 14 ♀	20/0	20/1	4.5	3/0	90%	90%	142%	95% <50 mL	—	40%	—
Creasey et al. [7]	16 ♂ 7 ♀	23/0	0/23	>1	8/2	78%	87%	—	70% <50 mL	Before 3/year After 2/year	100%	—
Vastenholt et al. [20]	32 ♂ 5 ♀	37/0	37/0	7.2	—	87%	57%	—	—	—	60%	65% (0% of ♂ used stimulation for sexual intercourse)
Hamel et al. [9]	4 ♂	4/0	0/4	—	—	100%	75%	—	100% <50 mL	—	50%	75% (0% of ♂ used stimulation for sexual intercourse)
Kutzenberger et al. [10]	440 ♂+♀	Almost all intradural	Almost all intradural	6.6	187/2	95%	83%	172%	—	Before 6.3/year After 1.2/year	91%	—

This overview includes several multicentre studies (∗∗) which include overlapping results with the reports of various single centre studies. Therefore, no accumulation of results is possible.

(−), unreported data or incomplete data for calculation; UTI, urinary tract infection.
